# Evaluation of Arsenic, Cadmium, Lead and Mercury Contamination in Over-the-Counter Available Dry Dog Foods With Different Animal Ingredients (Red Meat, Poultry, and Fish)

**DOI:** 10.3389/fvets.2018.00264

**Published:** 2018-10-25

**Authors:** Hyun-Tae Kim, John P. Loftus, Sabine Mann, Joseph J. Wakshlag

**Affiliations:** ^1^Department of Clinical Sciences, Cornell University College of Veterinary Medicine, Ithaca, NY, United States; ^2^Department of Population Medicine, Cornell University College of Veterinary Medicine, Ithaca, NY, United states

**Keywords:** dog food, ingredients, heavy metals, ICP-MS, pet food safety

## Abstract

**Objectives:** To examine the relative levels of heavy metals and arsenic content in commercial dog foods (arsenic, cadmium, lead and mercury) of 51 over-the-counter maintenance or all-life-stage dry dog foods. All products were chosen and segregated based on meat sources (fish, poultry, red-meat—17 products from each category) as animal protein sources being the primary contaminated ingredient due to bioaccumulation.

**Methods:** Inductively coupled plasma mass spectrometry (ICP-MS) was performed on products that were classified as fish, red meat (beef, pork, venison, bison) or poultry (chicken, turkey, duck) based. A non-Gaussian data distribution for each heavy metal within category distribution led to non-parametric statistical testing and median (range) descriptive statistics. Comparison to average human consumption based on mg/megacalorie (Mcal)was also examined.

**Results:** Based on caloric consumption, total arsenic and heavy metal consumption is higher in dogs than in humans; however chronic toxic exposure levels are highly unlikely. Fish-based diets had significantly higher arsenic, cadmium and mercury content than the poultry or red meat-based diets (*p* < 0.01). Red meat-based diets (beef, venison and bison) had higher lead concentrations than poultry and fish-based diets (*p* < 0.03).

**Clinical Significance:** Based on the findings, commercial dog foods appear to be safe for chronic consumption and concentrations of the heavy metals were dependent on primary protein sources. Overall, poultry-based diets had relatively lower heavy metal and arsenic content than red meat and fish-based diets. Despite the safety of most pet foods occasional outliers for lead render some concern for chronic exposure based on other species toxicity data and a lack of data in dogs.

## Introduction

Various ingredients, such as poultry, red meat, fish, grain, legumes, tubers and grains, are used as primary ingredients in contemporary pet food formulations. The ingredients and where they are geographically raised or grown will affect the degree of trace mineral inclusion (1, 2). Trace elements such as chromium, nickel, molybdenum and silica are essential for the growth and market preparation of domestic animals, but other elements such as arsenic, cadmium, lead, and mercury are metabolically unessential in general, and are known to cause serious health issues when exposed at sufficiently high concentrations (3–5). All of the trace minerals and heavy metals noted are not intended to be included in pet foods and higher consumption than environmentally normal likely originates from ingredients that were grown/exposed to environmental pollution and bioaccumulation over time (6, 7). Due to their constantly increasing prevalence in nature, arsenic, cadmium, and mercury bioaccumulation of our aquatic environments leads to fish being the most problematic ingredient in the food chain (8, 9). Lead, on the other hand, may be greater in (lead-shot) game meats or terrestrial animals exposed to lead from nearby structures contaminated with lead paints. Many of these terrestrial animals (such as bison and venison) are farm raised and longer lived leading to potential contamination and these protein sources which are increasingly utilized as novel protein sources by the pet food industry (10).

Excessive chronic intake of these undesirable heavy metals has been related to toxicities across many species, including dogs. Exposure to arsenic has been an observed cause of ulcerative dermatitis in dogs (11, 12), while cadmium influences male reproduction and pancreatic function in dogs (13, 14). Lead, as a neurotoxicant, localizes caudal to the optic chiasm causing functional disturbances of forebrain and cortical blindness, anemia, epileptic seizures, and bone sclerosis are other symptoms that can be seen in lead poisoned dogs (15). Mercury intoxication in dogs results in clinical signs throughout the gastrointestinal system including ulcerative stomatitis, glossitis, esophagitis, and hemorrhagic enteritis (16–18). Among various causes of noninfective myocarditis in dogs, arsenic, lead, and mercury intoxication can be potential causes (15). Moreover, all 4 heavy metals can also act as nephrotoxins causing chronic renal failure in dogs and cats (19, 20).

Since pets rely on commercial pet foods for daily energy and nutritional requirements, long-term feeding of the foods may lead to bio-accumulation depending on the product and level of contamination, yet overall absorption rates of the undesired metals are low, and heavy metal content in pet foods has yet to reported as the primary cause for the undesirable health issues discussed above (4). There are significant concerns for pet food safety due to the recurring exposure and lack of standards for these elements in pet foods, and there are no requirements for pet food manufacturers to test for heavy metal content. They are briefly mentioned in the National Research Council (NRC) and Association of American Feed Control Officials (AAFCO) publications due to their potential for toxicity, yet safe upper limits have not been proposed. There are safe upper limits of arsenic, cadmium, mercury and lead set for pet foods in European Union (EU) (2002/32/EC), but the regulation is largely based upon NRC recommendations (21). Moreover, EU standard regarding heavy metal inclusion is presented as dry matter weight of these elements (ppm). This method of reporting contamination of pet food does not take into consideration the differences in caloric content of different products and therefore does not provide an accurate potential for exposure, thus conversion to mg per common unit of consumption, which is the Mcal, is essential. As part of our examination of these heavy metals in pet foods we hypothesized that (1) fish based diets would be more highly contaminated with all heavy metals than red meat or poultry based products, (2) due to higher caloric consumption than humans per metabolic body weight that dogs would have a higher exposure than NRC published average human exposure and (3) average canine daily consumption would not reach known toxic exposure levels based on limited information in dogs. The aim or our study was to compare toxic heavy metal content (arsenic, cadmium, lead, and mercury) in several commercially available dry dog foods with different animal ingredient bases (red meat, poultry, and fish) using ICP-MS technology and these results were converted to consumption per megacalorie and then compared to human total diet studies of each trace element.

## Materials and methods

### Analyzed foods

Samples of 51 over-the-counter maintenance or all-life-stage dry dog foods (17 for each primary protein source—poultry, red meat, and fish) for healthy dogs were evaluated. Forty-seven dog foods were purchased from an online pet food retailer and 4 dog foods were obtained from a local grocery store (*n* = 1), a local pet food retailer (*n* = 1) and the Cornell Veterinary Medical Center (*n* = 2). The samples were all from different formulations regardless of they were from similar brands and each had a distinct production lot number. All samples were stored at room temperature and were submitted for heavy metal analysis within a week after acquisition and all foods were manufactured within a year of mill date. Foods examined came from the following company manufacturers; Nestle-Purina (4), Zignature (4), Natural Balance (3), Wellness (3), IAMS (2), Rachael Ray (2), FirstMate (2), Wild Calling (2), Nutro (2), Merrick (2), Blue Buffalo (2), Diamond (2), Annamaet (1), Holistic Select (1), EVO (1), KASIKS (1), Holistic Blend (1), Farmina (1), Dr. Tim's (1), Orijen (1), Acana (1), South Star (1), American Natural Premium (1), California Natural (1), Tuscan Natural (1), Pedigree (1), Royal Canin (1), American Journey (1), Go! (1), Instinct (1), Ol' Roy (1), AvoDerm (1), CANIDAE (1), Canine Caviar (1).

### Heavy metal analysis

All dog food samples were pulverized individually to avoid cross contamination. The samples were prepared in triplicate and put into plastic vessels using plastic utensils (5 g samples from each 100 g bag supplied) as part of submission to Eurofins Central Analytical Laboratories (Eurofins, New Orleans, LA, United States). Portions of the samples were digested with microwave-assisted nitric acid procedure and were dried in an oven at 40°C based on minor modifications to the methods of the International Organization of Agricultural Chemists (22). Concentrations of arsenic, cadmium, mercury and lead were determined via inductively coupled plasma mass spectrometry (ICP-MS) (Agilent 7500ce, Agilent Technologies, Santa Clara, CA, United States) coupled with Mira Mist nebulizer (Burgener Research, Mississauga, ON, Canada) measured in hydrogen mode (12). The lower limits of quantification for the analysis of the heavy metals were 0.001 mg/kg as fed for each heavy metal. Data output from ICP-MS was expressed in milligrams per kilogram and based on the energy density of each pet food obtained from manufacturers, the mean of triplicate analyses was divided by energy density (kcal/kg) of food to express heavy metal concentrations in mg/1,000 kcal. The intra-assay (1 sample tested 10 times) percent coefficient and inter-assay (5 samples tested on 3 separate days) percent coefficient of variations were below 5 and 7% respectively for all of the heavy metals examined. All food samples examined registered values above the lower limit of detection for each element.

### Normalization of heavy metal concentration to food caloric content

Metabolizable energy per kilogram food was collected from each pet food's label or via contact with the manufacturer. To compare heavy metal inclusions between products and with human daily intake, concentrations measured (mg mineral/kg food) were converted to mg/1,000 kcal Metabolizable energy (Mcal) of each metal. Median and range for heavy metal concentrations per Mcal in maintenance foods (*n* = 51) for dogs were subjectively compared with available toxicity and daily human intake information of each heavy metal (22).

### Statistical analysis

The non-Gaussian data distribution and lack of normality using Shapiro-Wilks testing led to non-parametric statistical testing using Kruskal Wallis analysis with Dunn's *post hoc* comparison across groups and descriptive statistics for each heavy metal using statistical software (JMP 12.0, Cary, NC, USA). Medians and ranges of each ingredient group were reported for all heavy metals. All calculated median mg/Mcal for average canine foods were compared to the mean human daily heavy metal intakes of a 79 kg male consuming 2,900 kcals per day adjusted to mg/Mcal intake (23).

## Results

### Arsenic

**Figure 1:**
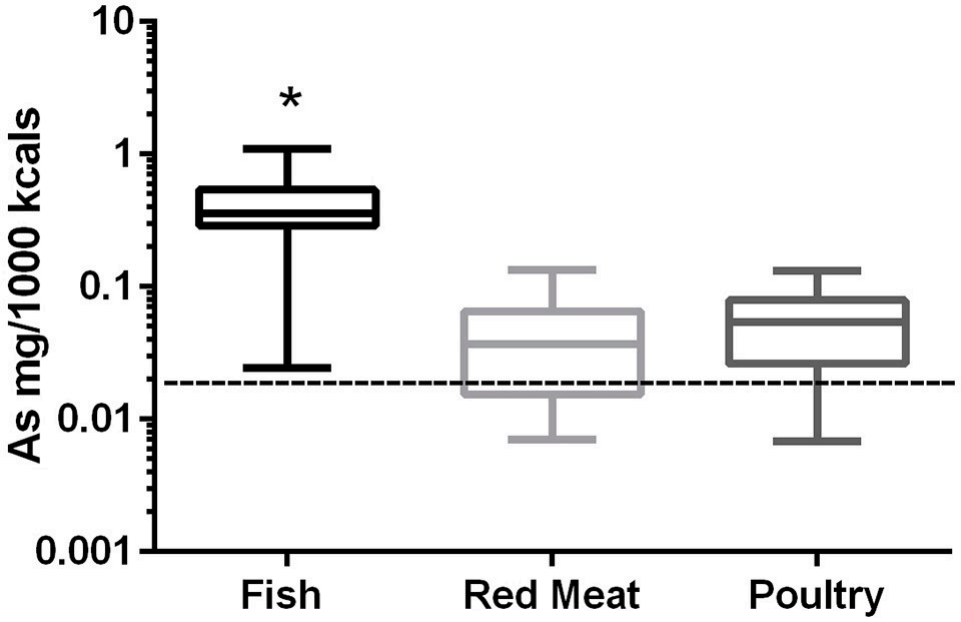
Box-and-whisker plots of the log arsenic concentrations in fish, poultry, and red meat-based dry dog foods (17 dog foods representing each for animal based protein source). Boxes represent the 25 and 75th percentiles, and whiskers represent the minimum and maximum. Solid lines within boxes represent the median values. Dashed lines indicate human intake (mg/Mcal) based on mean daily consumption. ^*^indicates a significant difference from both red meat and poultry based diets (*p* < 0.01).

In the 17 dog foods that had fish as the primary ingredient the median concentration of arsenic was 0.343 mg/Mcal (range: 0.025–1.104 mg/Mcal) (Figure 1), which was significantly higher than poultry-based and red meat-based foods whose medians were 0.054 mg/Mcal (range: 0.007–0.133 mg/Mcal) and 0.037 mg/Mcal (range: 0.007–0.134 mg/Mcal), respectively (*p* < 0.01). The median concentration of arsenic in all types of food tested exceeded the daily intake of humans (at least 2 fold) based on human diet studies (0.02 mg/Mcal) [Table 1; (24)]. The median arsenic containing fish-based diet had 16 fold higher arsenic concentration than daily mean human exposure in mg/Mcal, while the maximum arsenic containing fish-based diet was 55 fold higher than the median canine intake per Mcal, no fish-based diets were below human daily intake. Arsenic intake for poultry and red meat-based diets were lower than the average human intake in 3 and 5 diets analyzed, respectively, with all other products providing higher intakes per Mcal consumed.

**Table 1:** Median and ranges (mg/Mcal) of foods examined for heavy metal contamination (Arsenic, Cadmium, Mercury, and Lead) in commercial dog foods as compared to national research council assumptions of mean or range of daily human intake converted to mg/Mcal based on 2,900 kcal/day.

		**Median (range, mg/Mcal ME)**			
**Diets**	**No. of diets**	**Arsenic**	**Cadmium**	**Mercury**	**Lead**
Poultry	17	0.054	0.015	0.0008	0.037
		(0.007–0.133)	(0.005–0.063)	(0.0004–0.0023)	(0.019–0.305)
Red meat	17	0.037	0.013	0.0012	0.091
		(0.007–0.134)	(0.008–0.047)	(0.0004–0.0064)	(0.032–1.621)
Fish	17	0.343	0.027	0.0082	0.049
		(0.025–1.104)	(0.014–0.215)	(0.0010–0.0139)	(0.018–0.325)
Human intake		0.017–0.02	0.0034–0.0138	0.0016	0.006

### Cadmium

**Figure 2:**
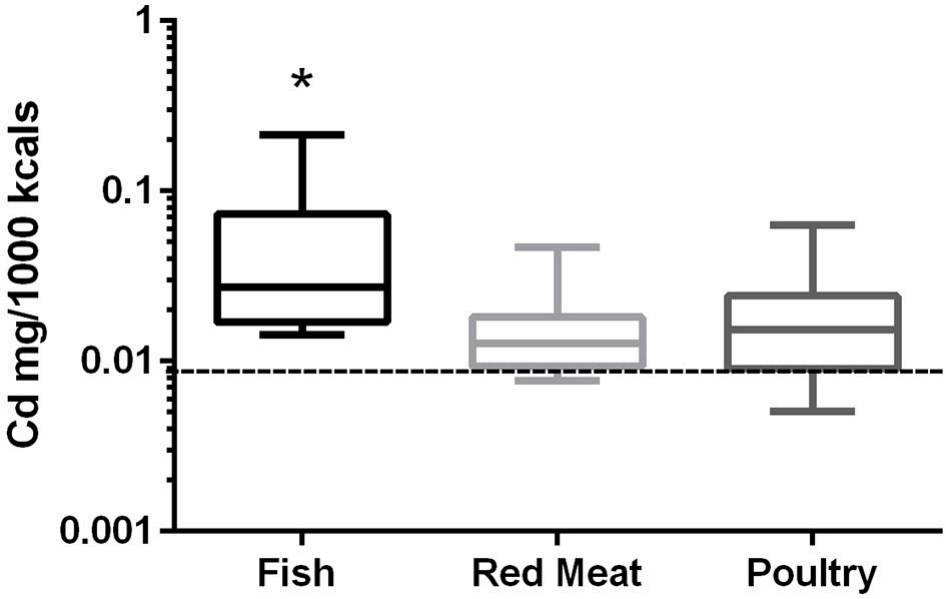
Box-and-whisker plots of the log cadmium concentrations in fish, poultry, and red meat-based dry dog foods (17 dog foods representing each for animal based protein source). Boxes represent the 25 and 75th percentiles, and whiskers represent the minimum and maximum. Solid lines within boxes represent the median values. Dashed lines indicate human intake (mg/Mcal) based on mean daily consumption. ^*^indicates a significant difference from both red meat and poultry based diets (*p* < 0.01).

In the 17 dog foods that had fish as the primary ingredient the median concentration of cadmium was 0.027 mg/Mcal (range: 0.014–0.215 mg/Mcal) (Figure 2), which was significantly higher than poultry-based and red meat-based foods whose medians were 0.015 mg/Mcal (range: 0.005–0.063 mg/Mcal) and 0.013 mg/Mcal (range: 0.008–0.047 mg/Mcal), respectively (*p* < 0.01). The median cadmium containing fish-based diet was 2 fold over maximal human daily cadmium exposure in North America and Europe (0.0034–0.0138 mg/Mcal; average 0.0086 mg/Mcal), and the maximum cadmium concentration found in fish-based diet was 15 fold higher [Table 1; (25)]. The poultry-based diet median exceeded the human maximal cadmium exposure per Mcal slightly, while the median red meat-based diets were within the range proving to be similar to human exposure per Mcal.

### Mercury

**Figure 3:**
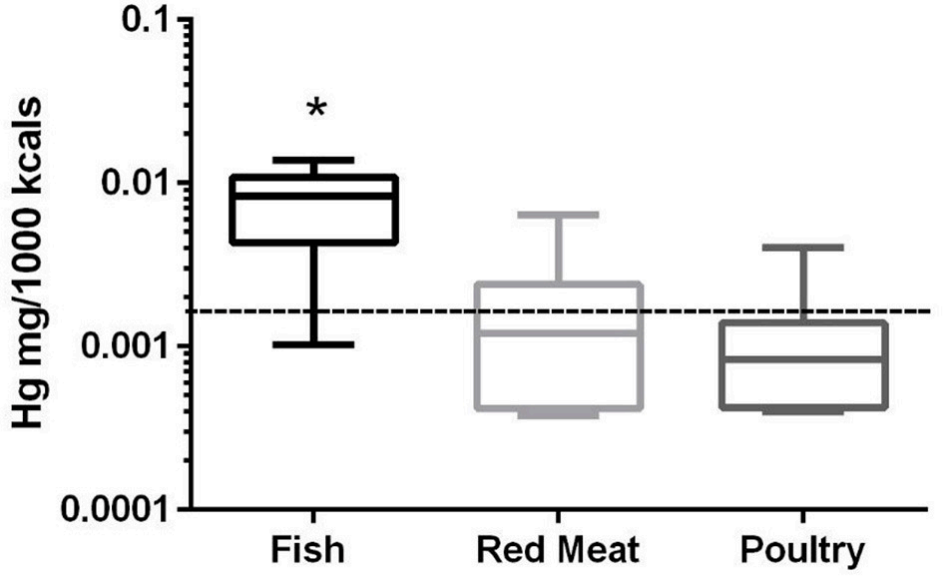
Box-and-whisker plots of the log mercury concentrations in fish, poultry, and red meat-based dry dog foods (17 dog foods representing each for animal based protein source). Boxes represent the 25 and 75th percentiles, and whiskers represent the minimum and maximum. Solid lines within boxes represent the median values. Dashed lines indicate human intake (mg/Mcal) based on mean daily consumption. ^*^indicated a significant difference from both red meat and poultry based diets (*p* < 0.01).

In the 17 dog foods that had fish as the primary ingredient the median concentration of mercury was 0.0082 mg/Mcal (range: 0.0010–0.0139 mg/Mcal) (Figure 3), which was significantly higher than poultry-based and red meat-based foods whose median were 0.0008 mg/Mcal (range: 0.0004–0.0023 mg/Mcal) and 0.0012 mg/Mcal (range: 0.0004–0.0064 mg/Mcal), respectively (*p* < 0.01). The median level of mercury in fish-based dog foods was 5 fold above maximum mercury intake in human total diet study (0.0016 mg/Mcal) [Table 1; (26)]. A fish-based diet with the highest mercury concentration was over 8 fold higher than the human daily intake. Medians of other types of diets were close to the range of average human mercury consumption per Mcal.

### Lead

**Figure 4:**
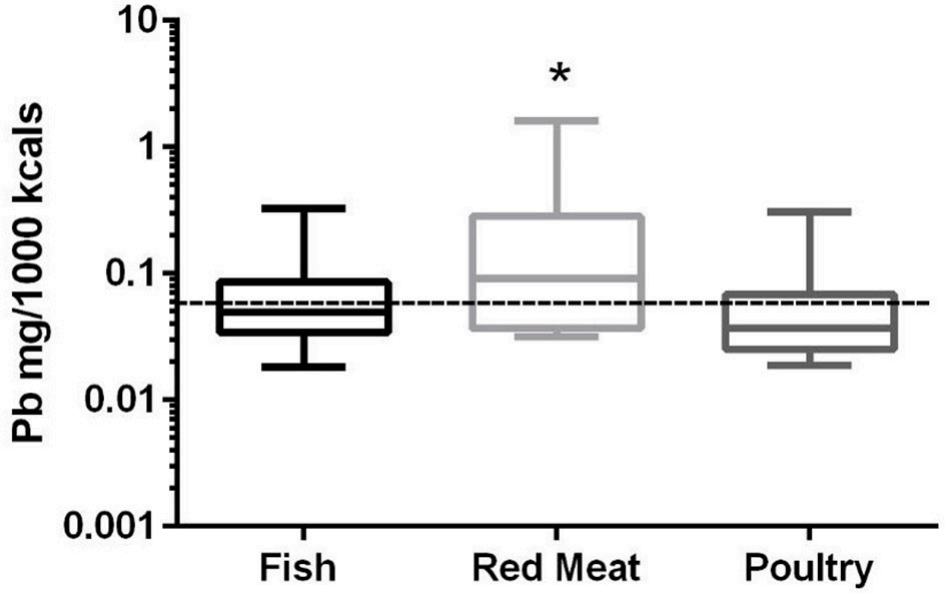
Box-and-whisker plots of the log lead concentrations in fish, poultry and red meat-based dry dog foods (17 dog foods representing each for animal based protein source). Boxes represent the 25 and 75th percentiles, and whiskers represent the minimum and maximum. Solid lines within boxes represent the median values. Dashed lines indicate human intake (mg/Mcal) based on mean daily consumption. ^*^indicates a significant difference from poultry but not fish based diets (*p* < 0.03).

In the 17 dog foods that had red meat as the primary ingredient the median concentration of lead was 0.091 mg/Mcal (range: 0.032–1.621 mg/Mcal) (Figure 4), which was significantly higher than poultry-based foods whose medians was 0.037 mg/Mcal (range: 0.019–0.305 mg/Mcal); while fish based median lead was 0.049 mg/Mcal (range: 0.018–0.325 mg/Mcal) and not significantly different than red-meat or poultry based diets (*p* = 0.03). The median concentration of lead in the red meat diet was over 15 fold higher than human daily lead intake (0.006 mg/Mcal) [Table 1; (27)]. The maximal lead concentration was also found in the red meat-based diets and was 270 fold greater than the human average daily intake per Mcal. Medians of poultry-based and fish-based diets were above the human dietary intake by 6 fold and 8 fold respectively.

## Discussion

There are few studies measuring the toxic heavy metal contamination of pet foods, and the extent of the elemental exposure compared to humans, and the possibility of intoxication from chronic consumption of the diets were not evaluated (6, 21, 28–30). Moreover, neither AAFCO nor NRC provides specific standards for these heavy metals since they are not essential nutrients (31). Our study results suggest that dogs may be exposed to higher levels of these undesired heavy metals than humans; however, this does not indicate a higher risk of toxicity from pet foods as consumption is well below known chronic toxic exposure levels.

Despite the well-known toxicity of arsenic, it may be an essential nutrient for reproduction in some species such as chickens, hamsters, goats, miniature pigs, and rats even though its requirement has not been well defined (32). Studies regarding arsenic metabolism propose that its function is related to modulation of DNA synthesis and trivalent arsenic (arsenite) is involved in methylation of histones, therefore regulating transcriptional activity (33, 34). However, the functions of arsenic have not been studied in dogs and most of the canine research has been focused on toxicity (19, 35). Exposure to a higher concentration of arsenic, especially inorganic forms, is related to myocarditis, dermatitis, and kidney and liver damage in dogs (11, 15, 36). Arsenic accumulates in the kidney and liver due to its water soluble nature, and higher arsenic concentrations found in urine are associated with chronic kidney disease (36). Administration of sodium arsenate (14.6 mg/kg BW) in dogs led to moderate to severe histologic degeneration (moderate glomerular sclerosis and severe acute tubular necrosis) in entire nephrons (37). Toxicity is dependent on the form of the element and inorganic arsenic is more toxic than organic forms and the form of arsenic in our study is not known. Organic arsenic in various marine fish species appears to be much higher than inorganic forms, and meats tend to accumulate organic forms, rather than inorganic forms (38). Moreover, dogs are less susceptible to inorganic arsenicals compared to humans as arsenic was used regularly as an anti-parasitic for dogs (39–41). A fish-based diet with the highest arsenic content (1.104 mg/Mcal) will be well below the dose provided by melarsomine (2.5 mg/kg body weight) and would be far lower than the lethal oral arsenic dose for other species (6–40 mg/kg) (4). However, the overall arsenic found in the analyzed diets leads to higher arsenic intake of dogs than humans. The impact of chronic arsenic exposure in dogs should be further assessed to evaluate potential health issues that occur in humans such as diabetes, hypertension, microvascular diseases and cancer (42–45).

Cadmium is an industrial pollutant that is a byproduct of lead and zinc production and utilization. In veterinary medicine, its intoxication is fairly uncommon and has limited significance especially in dogs (46, 47). Rather than acute poisoning, chronic and degenerative intoxications tend to be caused from cadmium contamination in foods. Sources contributing to higher cadmium content in pet foods include marine fish and organ meats. When ingested, cadmium induces the synthesis of metallothionein, a cysteine-rich protein that can transport cadmium to target organs such as kidney and liver and binds to metabolites in the tissues resulting in cellular injury (46, 48). To a lesser degree, free cadmium accumulates in the liver and reduces the synthesis of glutathione and promotes oxidative cellular damage resulting in extensive hepatocellular apoptosis and necrosis (49, 50). Cadmium accumulation can also interfere with vitamin D and calcium metabolism and can induce bone demineralization leading to bone loss in female dogs (51, 52). These toxicities are rare to non-existent since approximately 2% of ingested cadmium is absorbed enterically in most species, thus most of the ingested cadmium binds to enterocyte metallothionein and is sloughed as enterocytes mature and is eliminated in the feces (47). The median fish-based dog food containing cadmium in our study exceeded the upper range of human daily intake and the maximum concentration detected was 15 fold higher at approximately 0.2 mg/Mcal. However, a canine toxicity study done over 8 years in 10 dogs did not find histopathologic and radiographic abnormalities in dogs receiving approximately 10 mg cadmium per day (53), making toxicity from pet food exposure highly unlikely. In addition, there has yet to be a report of chronic consumption leading to toxicity, presumable due to the low bioavailability of this heavy metal.

Although mercury is a ubiquitous naturally occurring element in the environment, acute and chronic toxicity due to mercury is uncommon in domestic animals due to limited exposure (54). However, through bioaccumulation this metal concentrates in longer lived animals with saltwater fish having higher levels than most terrestrial animals due to exposure to high mercury levels from polluted water and accumulation through predation on other contaminated fish sources. Mercury can affect the nervous, renal, cardiovascular, hematopoietic and gastrointestinal systems (54). Neurologic signs caused by mercury include visual and motor dysfunction and the symptoms develop at lower doses than other organ toxicities from mercury intoxication. Eleven Beagles that were given 0.5 mg/kg/day methylmercury for a week began to exhibit changes in their behavior and developed impaired vision and motor coordination (55). As a neurotoxicant, methylmercury primarily impairs central nervous system at the cell bodies of the occipital cortex and the cerebellum and causes irreversible visual and motor deterioration (56). According to the results from this study, dogs eating fish-based diets have a higher possibility of being exposed to mercury than other animal ingredients; yet the highest consumption would be approximately 0.015 mg/Mcal which is far lower than the toxic dose of 0.5/mg/kg/day. Compared to human total diet studies, all of the fish-based diets in this study lead to higher daily mercury intake in dogs than humans, except one diet using farm-raised freshwater fish as the sole protein source which was lower. Most of the fish-based diets in this study use concentrated ingredients such as white-fish meals and they are likely to be 5 fold higher in mercury content than fresh fish (57). Moreover, >80% of mercury in fish meals are in the neurotoxic form, methylmercury (58). Among various forms of mercury, methylmercury is absorbed most efficiently by fish, but bioavailability of the mercury in fish meat is low considering a significant portion is bound to selenium (58). In dogs, the absorption rate of inorganic mercury is presumed to be approximately 40% (59). The presence of selenium in pet foods may further reduce the bioavailability of mercury in the food. Selenium is known to have a pronounced protective effect against methylmercury toxicity, but the concentration of selenium in pet foods required to impart protection is unknown (60, 61). Dietary phytate and fibers, which are common constituents of pet foods, are other potential inhibitors that alter bioavailability of methylmercury; but a rat experiment investigating fish meal ingestion did not show significant differences in the element's oral absorption rate with higher fiber diets (62).

Lead has no biological function and is considered toxic at certain levels of chronic exposure usually from environmental contamination. When absorbed, lead disrupts various biochemical reactions and cellular structures (63). Its toxicity causes gastrointestinal and neurologic signs depending on the affected species and duration of exposure. Abdominal pain and gastrointestinal signs such as diarrhea are the initial clinical manifestations when exposed to excessive amounts of lead, and neurologic signs such as depression, ataxia, seizure, and even death typically follow (63). As part of chronic exposure, lead also can accumulate in kidneys causing proximal tubular nephropathy (64). However, dogs given 10 mg Pb/kg diet for 2 years did not show deterioration of kidney function (65). Neurological signs, another major consequence of lead poisoning, can be seen in chronic lead administration of 0.005-0.01 mg/kg body weight/day in other species such as rats, monkeys and humans, but in dogs 40 weeks oral lead ingestion below 5 mg/kg BW/day did not lead to neuropathy or histological changes in the central nervous system (66, 67). Most of the lead toxicities occur via excessive oral lead ingestion and organic forms are absorbed better than other forms of lead compounds (63). Approximately 10% of lead ingested is absorbed and the alimental absorption rate of lead depends on the size of lead particle and nutrient composition of diets. Higher fat and lower calcium diets resulted in significantly greater gastrointestinal lead absorption in a canine lead toxicity study (68). The Centers for Disease Control and Prevention proposed toxic oral lead concentration of 300 mg/kg BW to cause death in dogs, but lifetime safe lead dose remains uncertain (69). The lead contents in the analyzed diets in this study were well below the dose used in the 2 years chronic exposure study suggesting a large margin of safety in pet foods; but overall lead intakes appear to be higher than the human daily lead intake. When examining all foods the highest amount being at 2 mg/Mcal does raise some concerns about chronic exposure in dogs since there are no canine studies examining lifetime or juvenile dog exposure from similar concentrations (70). In the case of young animals eating these types of diets during their growth, their higher absorption rate of up to 90% of oral lead intake could be problematic and their neural development could be affected based on other juvenile models. Therefore, puppy formulas should be tested for their lead content considering the potential effects in other species at similar concentrations as our highest product and our lack of knowledge in dogs, particularly puppies (71).

There are multiple limitation of our study that should be mentioned. Measurement of heavy metals in this study reflects the total concentration of the mineral and does not differentiate various toxic chemical forms of each mineral, and valency or organic/inorganic forms of these metals were not determined. Bioavailability differs depending on the mineral form and there may be variance of mineral bioavailability with species, and dogs may differ from other species regarding toxicity and chronic exposure. This study examined a single production run of each product and due to differences in sourcing and batch to batch differences in animal source proteins these heavy metal contaminants may fluctuate within a specific product. The pet food contamination may also be due to other sources such as water and machinery used during the extrusion, drying and enrobing process of the pet foods analyzed; however, the differences between animal based protein sources appears to be a major contributor. Human total dietary intake studies used to compare each mineral were done between 1987 and 2001 and may not reflect current daily intakes of the trace-minerals (24–27). However, this does not explain the significant difference between dog and human intakes which are likely related to the animal meal sources, the potentially higher protein diets that dogs are exposed to compared to humans as well as a slightly higher metabolic energy requirement of the average dog compared to the average person.

Based on results of this study, the primary protein ingredients of the dog foods influence concentrations of these heavy metal elements. Direct toxicity from the heavy metals in pet foods is unlikely to occur despite a paucity in reported data confirming this in the dog. However, it is conceivable that chronic exposure could contribute to diseases that occur in aged dogs eating the same product for many years, particularly lead since other species data suggests that lead exposure of 0.1 mg/kg body weight daily can lead to chronic neuropathy. Among the 3 types of protein sources evaluated in this study, poultry was lower in heavy metal and arsenic mineral contamination than red-meat or fish-based products. However, dogs consuming poultry-based diets will still be exposed to higher concentration of the trace elements than humans on a Mcal basis. This increased exposure is likely to be from higher metabolic energy requirement and the higher protein content of foods provide to the average dog compared to humans and the use of whole meat meals which can include organ meats (5). Although the products that we examined appear to be safe for consumption the lack of data in dogs and the potential for occasional products at the highest end of mg/Mcal contamination provide pause in suggesting that all dry pet foods are safe and consumers may want to solicit information form companies regarding heavy metal contamination before purchase.

## Author contributions

H-TK was responsible for data analysis and interpretation, drafting of the manuscript and approval of the submitted manuscript. SM was responsible for statistical data analysis and revision of the manuscript. JL and JW were responsible for the conception of study, supervised data collection, statistical analysis and manuscript editing.

### Conflict of interest statement

The authors declare that the research was conducted in the absence of any commercial or financial relationships that could be construed as a potential conflict of interest.

## Abbreviations

ICP-MS: Inductive coupled plasma-mass spectrometry
Mcal: megacalorie
AAFCO: American Association of Feed Control Officials
NRC: National Research Council
EU: European Union.
